# Molecular Mechanisms of Tebuconazole Affecting the Social Behavior and Reproduction of Zebrafish

**DOI:** 10.3390/ijerph20053928

**Published:** 2023-02-22

**Authors:** Wei Yan, Guangyu Li, Qiqi Lu, Jianjun Hou, Meiqi Pan, Maomin Peng, Xitian Peng, Hui Wan, Xixia Liu, Qin Wu

**Affiliations:** 1 Hubei Key Laboratory of Nutritional Quality and Safety of Agro-Products, Institute of Quality Standard and Testing Technology for Agro-Products, Hubei Academy of Agricultural Sciences, Wuhan 430064, China; 2 College of Fisheries, Huazhong Agricultural University, Wuhan 430070, China; 3 Hubei Key Laboratory of Edible Wild Plants Conservation and Utilization, Hubei Normal University, Huangshi 435002, China; 4 Huangshi Key Laboratory of Lake Biodiversity and Environmental Conservation, Hubei Normal University, Huangshi 435002, China; 5 Hubei Engineering Research Center of Special Wild Vegetables Breeding and Comprehensive Utilization Technology, Huangshi 435002, China

**Keywords:** tebuconazole, reproductive toxicity, social behavior, HPG axis, zebrafiah, mechanism

## Abstract

The aim of this study was to explore the underlying mechanism of adverse effects caused by tebuconazole (TEB) on the reproduction of aquatic organisms In the present study, in order to explore the effects of TEB on reproduction, four-month-old zebrafish were exposed to TEB (0, DMSO, 0.4 mg/L, 0.8 mg/L, and 1.6 mg/L) for 21 days. After exposure, the accumulations of TEB in gonads were observed and the cumulative egg production was evidently decreased. The decline of fertilization rate in F1 embryos was also observed. Then the changes in sperm motility and histomorphology of gonads were discovered, evaluating that TEB had adverse effects on gonadal development. Additionally, we also found the alternations of social behavior, 17β-estradiol (E2) level, and testosterone (T) level. Furthermore, the expression levels of genes involved in the hypothalamic-pituitary-gonadal (HPG) axis and social behavior were remarkably altered. Taken together, it could be concluded that TEB affected the egg production and fertilization rate by interfering with gonadal development, sex hormone secretion, and social behavior, which were eventually attributed to the disruption of the expressions of genes associated with the HPG axis and social behavior. This study provides a new perspective to understanding the mechanism of TEB-induced reproductive toxicity.

## 1. Introduction

Tebuconazole (TEB) is one of the most efficient and broad-spectrum 1,2,4-triazole fungicides, which can treat multiple fungal diseases in rice, wheat, peanuts, and vegetables [1]. Because of its high quality and widespread use, TEB can enter different environmental media through the natural water cycle, such as air, water, and soil [2]. The degradation half-life (time required to reach 50% degradation) of TEB in soil and water (25 °C) reached 216.6 days and 180 days [1,3]. Owing to its stable nature and high residue, TEB can be frequently detected in the natural environment. The concentration of TEB detected in surface water had reached 0.6–200 μg/L [4,5]. The maximum concentrations of TEB reported in agricultural runoff of Europe were 81 μg/L [6]. As a matter of fact, it was reported that TEB could accumulate in the organisms living in these environments [1,4]. For instance, the highest residual amount of TEB in brown rice was 0.9 mg/kg and the content of TEB in the muscles of fish (*Cyprinus carpio*) was 23.8 to 39.9 µg/kg [7,8]. There is even a study showing that TEB can be detected in the human body. Mercadante et al. [9] found that the metabolites of TEB detected in the urine of agricultural workers ranged from 3 to 473 μg/L. By this token, TEB, an aquatic pollutant of emerging concern, will pose a potential threat to the health of aquatic organisms and humans.

TEB was reported to induce multiple toxic effects on organisms living in these environments, mainly including developmental toxicity, hepatotoxicity, immunotoxicity, neurotoxicity, and reproductive toxicity [10,11,12,13,14]. It is well demonstrated that TEB is an endocrine disruptor. Numerous experimental data indicated that it might cause adverse effects on the reproduction of multiple species (eg. rat, earthworm, bird, *Xenopu laevis*, zebrafish). In male rats, TEB exhibits anti-androgen activity, leading to the decline of testosterone (T) levels in the offspring and interfering the sexual differentiation [15]. In addition, the egg production of birds that were fed with the seeds treated with TEB was decreased. Meanwhile, the 17β-estradiol (E2) level in the plasma of these exposed birds was notably reduced and the genes encoding key enzymes related to the biosynthesis of sterols and steroid hormones were also affected [16]. In earthworms, TEB disrupted the earthworm’s reproductive through the AMP pathway [17]. Concerning amphibians, the concentrations of E2 of the plasma in *Xenopus laevis* were greatly reduced after exposure to TEB for 27 days [10]. Regarding zebrafish, TEB could decrease the fecundity of zebrafish by interfering with the synthesis of steroid hormones [11]. Comprehensive the above literature, it can be concluded that TEB has been shown to have a negative impact on the reproduction of organisms by impairing the development of gonads and the levels of sex hormones.

Apart from hormone levels and reproductive parameters, growing evidence has shown that behaviors are critical for zebrafish reproduction [18,19,20]. A large number of studies have confirmed if normal patterns of reproductive behaviors were disrupted, reproductive success would be seriously impaired [21,22,23]. However, in recent years, some interesting studies have focused on non-reproductive behaviors which indicate that non-reproductive behaviors are key components of reproductive functions because they are essential for successful fertilization [19,24]. In teleosts, reproduction is not only dependent on the occurrence of reproductive behaviors, but also closely related to other non-reproductive behaviors, such as social behavior, and swimming behavior [19]. Social behaviors, which are key components of reproduction, provide many mating opportunities to conspecifics and reflect the mating intention, eventually interfering with mating behavior [18]. Meanwhile, swimming behavior reflects the locomotor activity of fish, which can maintain a series of normal social activities [19]. Therefore, these non-reproductive behaviors play vital roles in achieving the correct and effective reproductive behaviors, which has been verified in fish. In turquoise killifish, the sociability of the males exposed to fluoxetine was enhanced, contributing to the increase of mating frequency and reproductive output in fish populations [24]. Similarly, the male mosquitofish showed less intimacy and mating interest towards the gestodene-exposed females, indicating the time spent on attending, following, and mating behaviors decreased [25]. According to the above research, it is clear that the social interaction of female-male influences the mating behavior of fish. Now a growing stream of research suggests that TEB is capable of altering the behavioral responses of fish [26,27]. After being exposed to the TEB, the male zebrafish lost coordination of movements and resting stage at the bottom of the tank [27]. In tilapia, the behavior responses were significantly affected by TEB [26]. Integrating the above research, TEB has adverse effects on fish behavior. Hence, it is reasonable to speculate that TEB might induce reproductive toxicity by affecting social behavior in zebrafish.

For the sake of exploring the potential mechanism of reproductive toxicity induced by TEB, zebrafish were employed as the experimental animal in this study. We first examined the fecundity, sperm motility, and fertilization rate to assess the effect of TEB on reproduction. Meanwhile, gonadal histopathology, social behaviors, and contents of sex hormones were detected. In order to further elucidate the molecular mechanism of TEB-induced reproductive toxicity, the expression levels of genes associated with the HPG axis and social behavior were analyzed. This study will enrich the toxic mechanism of TEB from a new perspective.

## 2. Materials and Methods

### 2.1. Chemicals Used in This Study

TEB (CAS 107534-96-3, 99% purity) was purchased from Sigma-Aldrich. The TRIzol reagent was obtained from Invitrogen. PrimeScript^®^ RT Reagent Kits and SYBR^®^ Green PCR kits were bought from TakaRa (Dalian, China). Dimethyl sulfoxide (DMSO, Fisher Scientific, Fair Lawn, NJ, USA) was used as a solvent. All the chemicals used in this study were of analytical grade.

### 2.2. Zebrafish Maintenance and Experimental Design

Four-month-old zebrafish (*Danio rerio*) of the wild type (AB strain) were purchased from a commercial supplier. All fishes were housed in a fish breeding room with a light/dark cycle of 14/10 h. Charcoal-filtered tap water used in the fish breeding room was kept at 27 ± 0.5 °C. The relative humidity of the air was held at about 50%. The fish were fed at least for 14 d to adapt to the environment and each tank contains 15 females and 15 males. The mortality of zebrafish in this study was less than 5%. Culturing and breeding of adult fish were according to the described methods in OECD test guideline 229, which were fed with newly hatched brine shrimp (*Artemia nauplii*) three times a day in a quantity that was consumed within 5 min [28].

TEB was dissolved in DMSO, the solvent control group and exposure groups received 0.01% (*v*/*v*) DMSO. According to LC_50_ (median lethal concentration, 96 h) of TEB [27,29], the concentrations of TEB were set at 0, 0.4, 0.8, and 1.6 mg/L. The exposure solution with 0.01% DMSO was regarded as the solvent control group. There were three replicates for each group. Before the exposure experiment, in order to eliminate the influence of the breeding environment on spawning, the fecundity of fish (three male fish and three female fish) that were randomly chosen from each tank were tested by recording the number of eggs for 14 d. During the exposure duration, the six fish were paired every night in each tank for 21 d. The number of egg were counted in next morning after approximately 30 min of lighting. After counting, the cumulative production of eggs was computed. Then embryos were cultured in fresh water and the fertilization rate was also counted. The exposure water was renewed with the same fresh solution every two days which has been verified that the frequency of water exchange can maintain the concentration of toxin as the target concentration [11]. At the end of the exposure, the length and the total weight of each fish were recorded. After being taken blood, each fish was dissected to obtain the brain, liver, and gonad. Then brain-somatic index (BSI, brain weight × 100/body weight), hepatosomatic somatic index (HSI, liver weight × 100/body weight), and gonad somatic index (GSI, gonad weight×100/body weight) were calculated.

### 2.3. Contents of TEB in Gonads and Exposure to Water

The ovary and testis of 10 fish were separately collected to detect the accumulations of TEB in the gonads of zebrafish after exposure. Meanwhile, the contents of TEB in exposure water were also detected. The quantification method of TEB was according to Li et al. [11] and had been partially improved, which was described in Supporting Information (Test S1).

### 2.4. Measure of Sperm Motility

On the last day of the exposure experiment, the fish were paired but the partition in the spawning box was not removed in next morning. We collected semen by pressing the abdomen of males per replicate (*n* = 10, each concentration). Then an equal amount of semen was stored in Hank’s solution for excluding the effect of sperm concentration on sperm motility. The activated sperm was determined in the HT CASAII animal system, the measured concentration of sperm was between 200–300 sperm per observation field (4× microscope) to avoid being too large or too small. The experiments were carried out at room temperature (25 ± 1 °C). We recorded three classical metrics of sperm motility, including average movement rate (VAP), linear movement rate (VSL), and curve movement rate (VCL) by using the Computer-Assisted Sperm Analysis System (CASA).

### 2.5. Histological Examination

Histological analysis was conducted according to the methods previously described in [19]. The gonad tissues of fish (*n* = 6, each concentration) were fixed in Bouin’s solution for 24 h and then dehydrated in graded ethanol. After being embedded in paraffin, samples were sliced into sagittal sections (5 µm). Specimens were sealed with neutral gum after being stained by hematoxylin and eosin. The stained gonads can be observed by using a microscope (Soptop EX31, Sunny, Ningbo, China). The developmental stages of spermatocytes were classified into spermatogonia (SG), spermatocytes (SC), and spermatids (ST). Meanwhile, the development of oocytes was examined and classified into four stages: primary oocyte (PO), cortical alveolar oocyte (CAO), early vitellogenic oocyte (EVO), and late/mature oocyte (LO). The percentage of ovarian follicles in each developmental stage was expressed as a ratio of the number of corresponding ovarian follicles occupying the total follicles. The percentage of sperm cells at each stage was expressed as the ratio of the total sperm area occupied by the corresponding sperm cells.

### 2.6. Behavior Tests

After exposure for 21 days, a DanioVision system accompanied by EthoVision XT computer tracking software 15 (Noldus Information Technology) was utilized to detect swimming speed and distance in zebrafish. The testing method was according to the previous study [30]. In detail, each group was placed individually in water tanks (10 × 12.5 × 15 cm, high × wide × long) with 1 L water, and their swimming behavior was recorded (*n* = 16, each concentration). To assess social behaviors, fish were placed in water tanks (10 × 10 × 20 cm, high × wide × long) at a rearing density of a pair of fish, while fish from all exposed groups were examined (*n* = 16, each concentration). The distance threshold was set to 1.5 cm according to Wu et al. [30]. Two fish were thought to be in contact when a distance was less than 1.5 cm. Each pair of fish was recorded for 10 min. Using a video tracking system with the OpenOfficeOrg 2.4 software can analyze the number of contacts between fish and the time spent in the contact.

### 2.7. Quantification of Sex Hormones

Two sex hormones, E2 and T, were measured in the blood plasma of male and female fish. The method was referred to published protocols [31,32]. The blood of fish in each tank was collected. After taking a 5 μL plasma sample in the sterilized glass tube, and the plasma sample was diluted with 400 μL ultrapure water, then 2 mL ethyl ether was added to the diluted sample. The mix was centrifuged at 3000 r/min at 4 °C for 10 min. After mixing, the upper organic phase was absorbed into a new sterilized glass tube. The sample was repeated extraction twice according to the above steps. The collected organic phase was placed in the same dilution, which slowly dried the liquid with a nitrogen blower. Firstly, adding 60 μL buffer in the same dilution to again dissolve, which could be used in the determination of hormones. Then, E2 and T levels of plasma in zebrafish were examined by using the corresponding ELISA kits (Cayman Chemical Company, Ann Arbor, MI, USA), the detection limits of which were 15 and 6 pg/mL.

### 2.8. RNA Extraction and Real-Time Quantitative PCR (RT-qPCR) Analysis

Total RNA was extracted from samples of brains, livers, ovaries, and testes by using TRIzol, and their quality was evaluated by a spectrophotometer. RT-qPCR analysis was carried out as described previously [30]. Briefly, 1 μg total RNA was used to synthesize cDNA by use of PrimeScript™ RT reagent Kits. RT-qPCR amplifications with SYBR™ Premix Ex Taq™ reagent Kits and primers were used to quantify the mRNA of all target genes. Thermocycling protocols were as follows: 30 s at 95 °C, 40 cycles of 5 s at 95 °C, and 30 s at 60 °C. Expression of *β-action* was stable and used as a housekeeping gene. Expressions of the detected genes were normalized to *β-action* by use of the 2^−ΔΔCt^ method, which was not significantly different between control and exposure groups.

### 2.9. Statistical Analyses

Statistical analyses were conducted by using SPSS 17.0 software and data were expressed as mean ± standard error of the mean (SEM). The Kolmogorov-Smirnov test and Levene’s test were employed to examine the normality of the data and the homogeneity of variances. Statistically significant differences were determined by the use of one-way analysis of variance (ANOVA) with Tukey’s multiple range test. Significant differences were taken as *p* < 0.05.

## 3. Results

### 3.1. Accumulation of TEB in Gonads and Exposure to Water

The accumulations of TEB in gonads and water were shown in (Table 1). From the results, the content of TEB in the ovary from exposure groups (0.4 mg/L, 0.8 mg/L, and 1.6 mg/L) were 5.65 ± 0.06, 8.87 ± 0.07, and 14.72 ± 0.04 µg/g·WW (wet weight), respectively. Meanwhile, the accumulations of TEB in testis were 5.78 ± 0.05 (0.4 mg/L group), 7.95 ± 0.09 (0.8 mg/L group), and 13.83 ± 0.07 µg/g·WW (1.6 mg/L group). The recovery rates in the ovary and testis were 90.56 ± 11.23% and 89.23 ± 5.83%. The results showed the TEB concentrations of exposure water were stable and relatively close to the target concentrations.

### 3.2. Egg Production, Fertilization Rate, and Somatic Indexes

**Figure 1:**
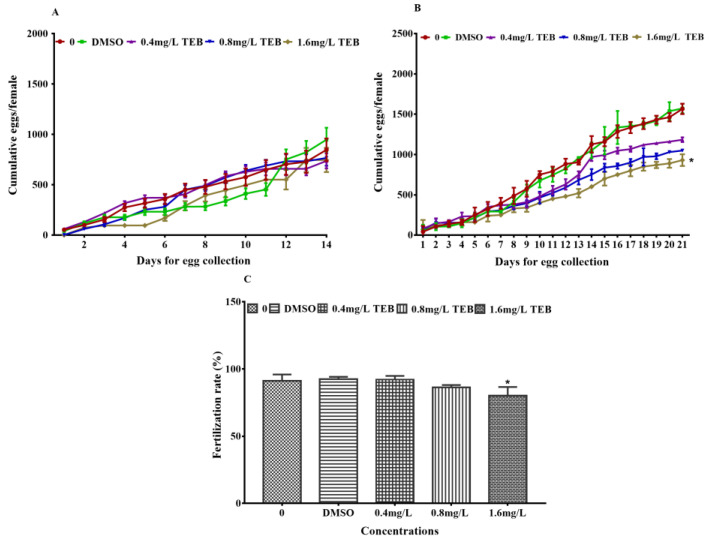
Cumulative egg production of zebrafish in 14 days before exposure (**A**) and 21 days (**B**) during exposure (3 pairs of fish per replicate, 3 replicates). (**C**) Fertilization rates of F1 embryos from the parents exposed to TEB (200 larvae per replicate, 4 replicates). Data are expressed as the mean ± SEM of three independent experiments. Asterisks in the figures were regarded as statistical differences between the exposure groups and control group (Tukey: * *p* < 0.05).

The mean cumulative egg production during the pre-exposure (14 days) and exposure periods (21 days) were shown in Figure 1. There was no significant difference in egg production among all groups during the pre-exposure period. Compared to the control, the cumulative egg production in the 1.6 mg/L TEB group was significantly reduced by 46%, while it was not remarkably changed in other exposure groups. Meanwhile, the fertilization rate of F1 embryos was notably decreased only in the 1.6 mg/L TEB group (Figure 1C).

Somatic indexes are important indicators to evaluate fish reproduction shown in Supporting Information (Table S1). No significant effect was observed in the BSI of zebrafish in all the exposure groups. However, the HSI was notably increased in female fish from the 1.6 mg/L TEB group and in male fish from all the exposure groups. GSI remarkably declined in females of the 1.6 mg/L TEB group, while there was no significant difference in males, which demonstrated that females were more sensitive to TEB than males.

### 3.3. Histological Analysis of Gonads

**Figure 2:**
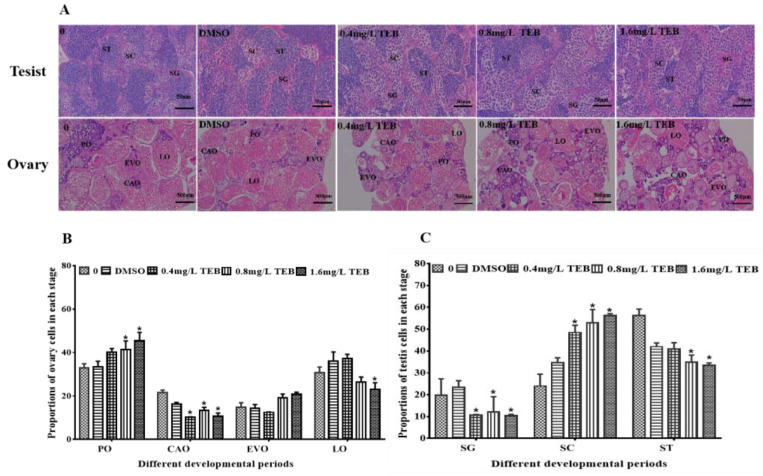
Effects of TEB on the gonadal development of zebrafish. (**A**) sections of ovary and testis stained by H&E (mean ± SEM, *n* = 6). Bars represent 50 µm (900×) and 500 µm (90×). Percentages of primary oocytes (PO), cortical oocytes (CAO), early vitellogenic oocytes (EVO), late/mature oocytes (LO), spermatogonia (SG), spermatocytes (SC), and spermatids (ST) were shown in (**B**,**C**). Asterisks in the figures were regarded as statistical differences between the exposure groups and control group (Tukey: * *p* < 0.05).

In this study, the development of the ovary and testis in adult zebrafish was inhibited. The proportions of various cells in the DMSO group were not remarkably different from the blank group, which indicated that the solvent has little effect on the development of the gonads. For female fish, the PO, CAO, EVO, and LO in the control groups and TEB groups showed normal characteristics (Figure 2A). The proportion of PO in 0.8 mg/L and 1.6 mg/L TEB groups significantly increased by 25.2% and 38.0%. The proportion of CAO was notably decreased in all exposure groups (52.6%, 0.4 mg/L TEB group; 37.9%, 0.8 mg/L TEB group; 50.1%, 1.6 mg/L TEB group) (Figure 2B). The proportion of LO was significantly reduced by 25.5% in the 1.6 mg/L TEB group. Similarly, the percentage of SG, SC, and ST in males was also detected (Figure 2C). Compared with the control group, the percentages of SG in all the exposure groups were notably decreased (46.0%, 0.4 mg/L TEB group; 38.9%, 0.8 mg/L TEB group; 47.2%, 1.6 mg/L TEB group), while the percentages of SC in 0.4 mg/L, 0.8 mg/L, and 1.6 mg/L TEB groups were significantly increased by 102.1%, 120.9%, and 134.3%. However, the percentages of ST were notably decreased in the 0.8 mg/L (37.8%) and 1.6 mg/L TEB group (40.7%).

### 3.4. Sperm Motility of Male Zebrafish

**Figure 3:**
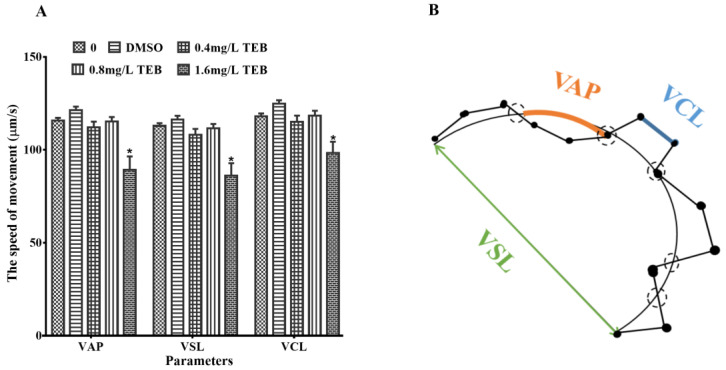
(**A**) Comparison of forward sperm motility movement parameters between exposure and control groups. VAP, average path speed; VSL, Line motion speed; VCL, curve motion speed (mean ± SEM, *n* = 10). (**B**) Schematic representation of sperm motility patterns according to a computer-assisted sperm analysis system. Asterisks in the figures were regarded as statistical differences between the exposure groups and control group (Tukey: * *p* < 0.05).

It was shown that the three motor parameters of VCL, VSL, and VAP have a stronger association with the rate of fertilization compared with the other parameters. The three motor parameters were not dramatically affected in the solvent control group compared to the blank control. Nevertheless, the three indicators of sperm swimming velocity (VAP, VSL, and VCL) were remarkably reduced by 19.9%, 21.6%, and 13.7% in the 1.6 mg/L TEB group, respectively (*p* < 0.05) (Figure 3). And VAP, VSL, and VCL of sperms in other exposure groups were not notably altered.

### 3.5. Behavior of Zebrafish

**Figure 4:**
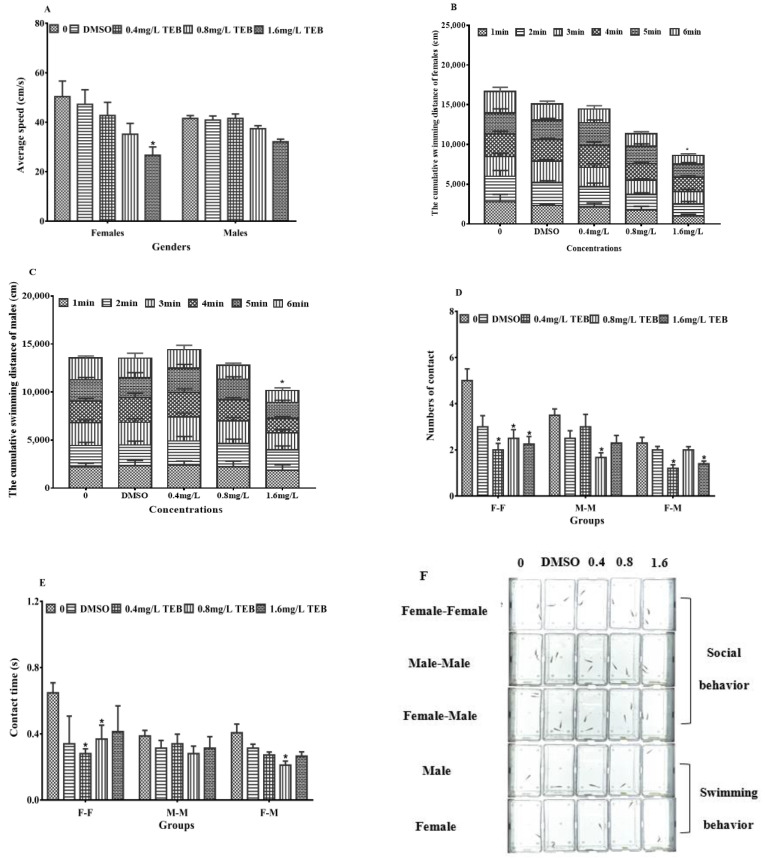
(**A**) Mean swimming speed of female and male zebrafish after exposure for 21 days (mean ± SEM, *n* = 16). Cumulative swimming distance of female (**B**) and male (**C**) zebrafish after exposure to TEB for 21 days (mean ± SEM, *n* = 16). Social behaviors of females and males (mean ± SEM, *n* = 16). (**D**) numbers of contacts, (**E**) contact time, F-F, female and female; M-M, male and male; F-M, female and male. (**F**) Real-time pictures of behavior detection. Asterisks in the figures were regarded as statistical differences between the exposure groups and control group (Tukey: * *p* < 0.05).

The average velocity of female zebrafish in the highest concentration group was significantly decreased by 48.8% (*p* < 0.05) (Figure 4A). The average velocity of males in exposure groups was not remarkably affected, while it showed a downward trend. The cumulative swimming distances of zebrafish were consistent with changes in the mean velocity, and remarkable reductions of 48.3% and 25.0% were observed in the females and males from the 1.6 mg/L TEB group (Figure 4B,C). Changes in swimming movement in females were greater than that in males, indicating that females might be more sensitive to TEB.

The effects of TEB on the social performance of zebrafish were shown in Figure 4D,E. In female-male, the time of contact with interactions was notably decreased by 48.1% in the 0.8 mg/L TEB group. The number of contacts significantly decreased by 47.8% and 39.1% in the 0.4 mg/L and 1.6 mg/L TEB groups. In male-male, the number of contacts was remarkably decreased by 52.4% in the 0.8 mg/L TEB group, while there was no notable change in the time of contacts. In addition, the time and number of contacts in female-female were affected after exposure to TEB. The contact time was remarkably decreased by 56.8% and 43.2% in the 0.4 mg/L and 0.8 mg/L TEB groups. In addition, the contact number was also significantly decreased in all the exposure groups (60.0%, 0.4 mg/L TEB group; 50.0%, 0.8 mg/L TEB group; 55.0%, 1.6 mg/L TEB group).

### 3.6. Plasma Sex Hormones

**Figure 5:**
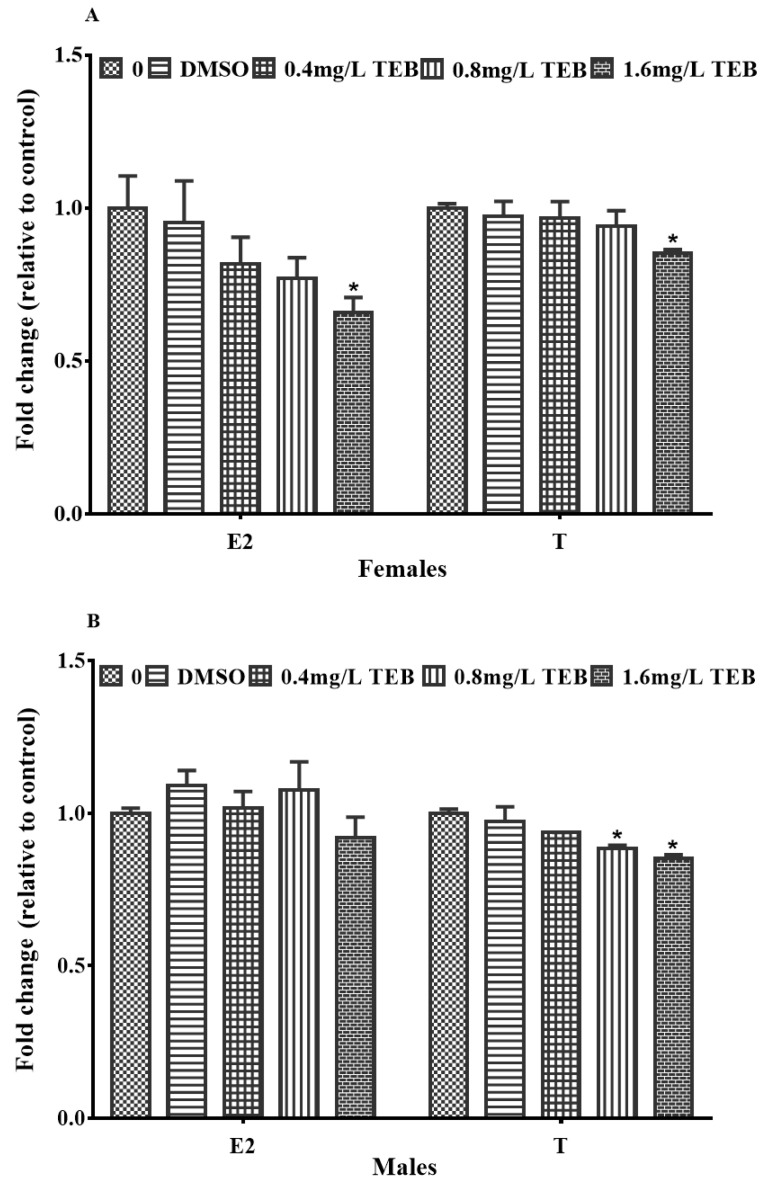
Influence of TEB on the levels of E2 and T in female (**A**) and male (**B**) zebrafish (mean ± SEM, five fish per replicate, 4 replicates). Asterisks in the figures were regarded as statistical differences between the exposure groups and control group (Tukey: * *p* < 0.05).

The sex hormone levels in both females and males were altered after exposure to TEB. In females, the plasma E2 and T levels were observably reduced by 34.1% and 14.7% in the 1.6 mg/L TEB group (Figure 5A). In males, the concentration of T was notably decreased by 11.6% and 14.7% after exposure to 0.8 mg/L and 1.6 mg/L TEB (Figure 5B). However, the E2 level was not evidently affected.

### 3.7. The Expression Levels of Genes Related to the HPG Axis and Social Behavior

**Figure 6:**
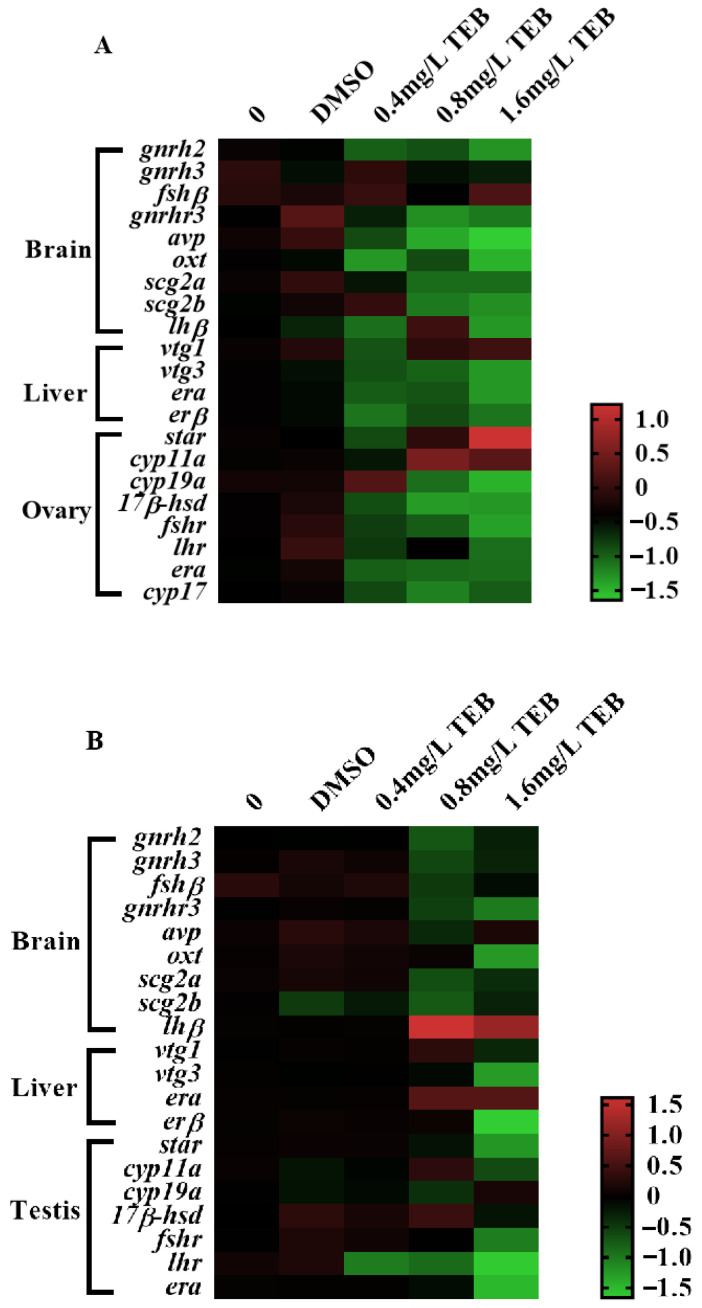
Heat map of gene expressions associated with social behavior and HPG axis of female (**A**) and male (**B**) zebrafish (mean ± SEM, *n* = 6), and the values in the result were derived from the fold change of gene expression converted by a log 2 as the bottom.

The expression levels of genes involved in the HPG axis and social behavior were evaluated in the present study after exposure to TEB for 21 days (Figure 6), the detailed expression levels of which were summarized in the Supporting Information (Tables S2 and S3). In the brain of females, the transcription levels of *gnrh2*, *oxt*, *scg2b*, and *lhβ* (1.36-, 1.73-, 1.18-, and 1.33-fold) in 1.6 mg/L TEB group were significantly down-regulated. The gene expression levels of *gnrhr3* (1.20- and 0.94-fold) and *avp* (1.72- and 2.31-fold) showed obvious declines in the 0.8 and 1.6 mg/L TEB groups. There were no notable changes in expression levels of the *gnrh3, fshβ,* and *scg2a* genes. In the female liver, the expression levels of the *vtg3* and *erα* genes were both decreased by 1.35-fold in the 1.6 mg/L TEB group. However, *erβ* and *vtg1* were not remarkably affected. In the ovary, the transcription of *star* (2.32-fold) involved in the steroidogenic pathway was prominently up-regulated after exposure to 1.6 mg/L TEB, while down-regulated transcriptions of *cyp19a, 17β-hsd*, and *fshr* (1.92-, 1.35-, and 1.46-fold) were observed. In addition, *cyp11a* was notably up-regulated in 0.8 (1.64-fold) and 1.6 mg/L (1.44-fold) TEB groups.

In male fish, *gnrhr3*, *oxt*, and *scg2b* genes in *the* brain (1.00-, 1.45-, and 0.68-fold) were significantly down-regulated after exposure to 1.6 mg/L TEB, whereas, the transcription of *lhβ* (2.26-fold) gene was remarkably up-regulated in 1.6 mg/L TEB group. There were no changes in expression levels of *gnrh2*, *gnrh3*, *fshβ*, *avp*, *scg2a*, and *scg2b* genes. In the liver, the notable down-regulation of *vtg3* and *erβ* (1.45- and 2.24-fold) was observed in 1.6 mg/L TEB. In testis, the *erα* and *fshr* (1.91- and 1.04-fold) genes were significantly down-regulated in the 1.6 mg/L TEB group. Additionally, the expression level of the *lhr* gene was notably decreased by 1.02-, 0.96-, and 2.38-fold in 0.4, 0.8, and 1.6 mg/L TEB groups, respectively.

## 4. Discussion

In the present study, the accumulation of TEB in gonads and the decrease of egg production in females were found after exposure to TEB, indicating TEB accumulation could evidently decline the reproductive capacity of zebrafish and show negative effects on the reproduction system of zebrafish which was in accordance with the previous study [11]. Meanwhile, the GSI of zebrafish after exposure was remarkably decreased. GSI is a parameter in toxicological studies and is considered to be related to egg production [31]. Judging from this, the alternation of GSI was consistent with the decrease in egg production. Additionally, the fertilization rate of F1 embryos was also significantly affected by TEB. Taking all these results together, it was indicated that the TEB-elicited reproductive toxicity through affecting the reproductive capacity of parental zebrafish and the fertilization rate of F1 embryos.

For the sake of exploring the underlying mechanism of the decreased egg production and fertilization rate, the development of gonads and sperm motility were examined. The development of gonads was inseparable from the normal formation of the germ cells, which eventually contributed to the reproductive success of zebrafish [33,34]. Through the results of histopathological examination in this study, it was found that the proportion of each cell type (CAO and LO in the ovary; SC and ST in the testis) at each stage were significantly decreased. Consistent with our results, Lu et al. [34] found that TEB could disrupt the development of gonads in *Caenorhabditis elegans*. A similar phenomenon was found in Hyla intermediate membrane larvae. The testis was hypoplastic and the seminal sac leaflets were barely recognizable after exposure to TEB [33]. Hence, all this evidence indicated TEB had an adverse effect on the development of gonads. And the abnormal development of gonads in zebrafish led to the reduction of GSI and egg production. Furthermore, a reduction in sperm viability was found in this study. There is evidence that sperm viability is closely related to reproductive success in zebrafish [35]. For example, long-term exposure to nonylphenol could inhibit the sperm motility of male zebrafish, ultimately leading to a decrease in the fertilization rate [36]. Chen et al. [22] also found this phenomenon in zebrafish. Additionally, sperm quality has interwovenness with the development of the testis. Thus, from the above evidence, it can be concluded that TEB exerted an interfering effect on gonadal development and function which contributed to the decrease in sperm viability, egg production, and fertilization rate.

There was growing evidence that confirmed that the synthesis and secretion disorders of steroid hormones (T and E2) and vitellogenin (VTG) could affect the process of gonads development [32,37]. Hence, we investigated the contents of T and E2 in the plasma of zebrafish as well as the variety in gene expressions of the HPG axis to explore how TEB exerted toxic effects on the development of gonads. T and E2 directly affect spermatogenesis and the proliferation of oocytes [37]. Afterward, after binding to estrogen receptors (ERs) in the liver, E2 could induce the generation of VTG which was an important precursor for the synthesis of vitellogenin, and then affect the development of oocytes [38]. It was reported that the HPG axis occupied an important position in the process of reproduction by regulating the synthesis and secretion of hormones (gonadotropin-releasing hormone (GnRH), gonadotropin (Follicle stimulating hormone (FSH), and lutein (LH)), VTG, and sex hormones (T and E2) in the endocrine system [39]. In this study, the levels of E2 and T in plasma were dramatically decreased in zebrafish after exposure to TEB. Meanwhile, TEB exposure altered transcription levels of these genes involved in the biosynthesis of the HPG axis (*gnrh2*, *lh*, *lhr*, *vtg3*, *er*, *star*, and *cyp19α* in females; *gnrhr3*, *lh*, *vtg3*, and *fshr* in males). As reported in previous studies, the delay of gonadal development caused by contaminants (tris (1,3-dichloro-2-propyl) phosphate; microcystin-LR; pyriproxyfen; bisphenol AF) was attributed to the changes in E2, T or VTG [39,40,41,42]. Equally, these phenomena were observed in pregnant Sprague-Dawley rats exposed to TEB for 10 days [43]. Hence, based on the above findings, it could be suggested that TEB might induce toxic effects on the gonadal development of zebrafish by inhibiting the synthesis of steroid hormones and interfering with the expression of genes along the HPG axis, ultimately contributing to the decline of egg production and sperm viability.

Apart from the normal development of the gonads, reproductive success in fish also depends on reproductive behaviors and non-reproductive behaviors [44,45]. Non-reproductive behaviors (such as social behavior, and swimming behavior) have been recognized as sensitive endpoints which are critical for successful reproduction [45]. Among these behaviors, social behaviors, are crucial components of reproduction, which are necessary for successful fertilization [46]. Besides fertilization, social behaviors between females and males (males rapidly swing their tail against the female side) trigger the spawning of females. Actually, in the process of mate choice and mating, social interaction also plays a vital role [47,48]. Therefore, alternations in social behavior may influence reproductive behaviors, causing a decrease in egg production. In this study, both the number and time of interaction in zebrafish declined dramatically, implying mating attempts were affected after exposure to TEB. Simultaneously, the swimming speed of zebrafish from exposure groups was notably slower than that of the control group. A decrease in social behavior may be associated with a decrease in motility. Consequently, combined with the above literature and the results in this study, there were reasons to consider that the effect of TEB on social behavior might contribute to the spawning and fertilization of the egg, eventually inducing reproductive toxicity in zebrafish. The social behaviors of fish are mainly controlled by the central nervous system (CNS) and the neuroendocrine system [21]. In the neuroendocrine system, neuropeptides synthesized and secreted in the hypothalamus may influence the social behavior of fish [49]. Among these neuropeptides, secretoneurin (SN) generated by the two secretogranin-2 (SCG2) subtype precursor proteins is a member of a peptide family that is the key to the modulation of social behaviors and reproductive [50]. Mutation of the scg2 genes reduced social sexual behavior, oviposition, and fertility in zebrafish [51]. Additionally, isotocin (OXT) and vasotocin (AVP) which are co-expressed with SCG2 are well-known regulators of social behavior [50,52]. A growing body of evidence supports that SCG2, OXT, and AVP are associated with social behavior [53,54]. Hence, for the sake of exploring the mechanisms of TEB affecting social behavior, we further examined the transcriptions of behavior-related genes (*scg2a, scg2b*, *oxt*, and *avp*). After exposure to TEB, *scg2b*, *avp*, and *oxt* genes in the brain were notably down-regulated, which was consistent with the previous studies [51]. Maruska et al. [55] also reported that the abnormality of social behavior was owing to the interruption of the expression levels of these hypothalamic neuropeptide genes or the activity of hypothalamic neurons, resulting in abnormal reproductive function. Meanwhile, in electric fish, SN and AVP were also confirmed to modulate social behavior [50]. There is also evidence that these hypothalamic neuropeptides (SN, OXT, and AVP) regulate social behaviors by regulating the HPG axis [53,54]. Consequently, TEB might influence the social behavior of zebrafish by interfering with the expression of genes involved in social behavior and the HPG axis, eventually affecting the reproduction of zebrafish.

## 5. Conclusions

Taken together with all the results, it could be concluded that TEB could affect the egg production and fertilization rate by interfering with gonadal development, sex hormone secretion, and social behavior, eventually leading to adverse effects on the reproduction of zebrafish. After further analysis, the reasons for the decline of fecundity and fertilization rate caused by TEB were the disruption of the expressions of genes associated with social behavior and the HPG axis. Therefore, our study provides a new perspective to understanding the mechanism of TEB-induced reproductive toxicity, contributing to the ecological risk evaluation of TEB.

## Figures and Tables

**Table 1:** Accumulation of TEB in goads and exposure water.

Groups	Ovary (µg/g WW)	Testis (µg/g WW)	Before Water Renewed	After Water Renewed
Control	ND	ND	ND	ND
Solvent Control	ND	ND	ND	ND
0.4 mg/L TEB	5.65 ± 0.06	5.78 ± 0.05	0.32 ± 0.02	0.39 ± 0.01
0.8 mg/L TEB	8.87 ± 0.07	7.95 ± 0.09	0.74 ± 0.04	0.79 ± 0.01
1.6 mg/L TEB	14.72 ± 0.04	13.83 ± 0.07	1.53 ± 0.01	1.58 ± 0.01

The values represent the mean ± SEM of 5 replicates. ND, Not detected. WW, Wet weight.

## Data Availability

All data and materials generated or analyzed in this study are included in this published article and its Supplementary Information.

## Supplementary Materials

The following supporting information can be downloaded at: https://www.mdpi.com/article/10.3390/ijerph20053928/s1. Test S1: Method of the quantification of TEB in gonads and exposure water; Table S1: Somatic indexes of zebrafish after exposure to TEB for 21 days; Table S2: Expression of the genes associated on social behavior and HPG axis of female zebrafish exposed to TEB; Table S3: Expression of the genes associated on social behavior and HPG axis of male zebrafish exposed to TEB; Table S4: The full name of the genes.
