# Supplementary material for: Molecular Mechanisms of Tebuconazole Affecting the Social Behavior and Reproduction of Zebrafish
Source: Int J Environ Res Public Health. 2023 Feb 22;20(5):3928. doi: 10.3390/ijerph20053928 (PMC10002025; doi:10.3390/ijerph20053928)
Supplement: Supplementary file 1 [file ijerph-20-03928-s001.zip › ijerph-2214209-supplementary.pdf]

Supporting Information

Molecular mechanisms of Tebuconazole affecting the social behavior and reproduction of zebrafish

Wei Yan[1], Guangyu Li[4], Qiqi Lu[2,3], Jianjun Hou[2,3], Meiqi Pan[4], Maoming Peng[1], Xitian Peng[1], Hui Wan[2,3], Xixia Liu[2,3], Qin Wu[2,3*]

[1]Institute of Quality Standard and Testing Technology for Agro-Products, Hubei Academy of Agricultural Sciences, Hubei Key Laboratory of Nutritional Quality and Safety of Agro-products, Wuhan, 430064, Hubei, China

[2]Hubei Key Laboratory of Edible Wild Plants Conservation and Utilization, Huangshi Key Laboratory of Lake Biodiversity and Environmental Conservation, Hubei Normal University, Huangshi, Hubei province, 435002, China

[3]Hubei Engineering Research Center of Special Wild Vegetables Breeding and Comprehensive Utilization Technology, Huangshi, Hubei province, 435002, China

[4]College of Fisheries, Huazhong Agricultural University, Wuhan 430070, China

***Author for correspondence:**

Qin Wu (Q. Wu) PhD
Hubei Key Laboratory of Edible Wild Plants Conservation and Utilization, Hubei Normal University
Huangshi 435002, China
Fax: 86-27-87389465
Email: wuqin20200106@163.com

Test S1. Method of the quantification of TEB in gonads and exposure water

Before examinations, the pretreatments of tissue samples and water samples were conducted. About 20 mL of test solution from the middle of the water of the test aquarium was taken, and filter it with 0.45 μm water system filter membrane to obtain the water samples. The pretreatment of water samples as followed: 1mL of water samples into a measuring cylinder with a stopper, then accurately adds 10 mL of acetonitrile, 1 g NaCl and 4 g $MgSO_4$, shakes for 1-2min for stratification. After standing for 30 min, take the upper organic phase and filter it through 0.2 μm to be determined by UHPLC-MS/MS injection. Meanwhile, gonad samples were dried with absorbent paper and weighted. Then put them into the mortar and weigh the same amount of NaCl for grinding. Then the powder was transferred to 50 mL EP tubes. 4g $MgSO_4$ and 5 mLacetonitrile were added to the tubes. After vortex vibration, the samples were centrifuged at 4000 rpm for 10 min. 5 mL 5% methyl alcohol aqueous solution (methanol: water=5:95, V/V) was used to dissolved the samples. After purified, the water samples and gonad samples were filter it through 0.2 μm to be determined by UHPLC-MS/MS injection.

Table S1. Somatic indexes of zebrafish after exposure to TEB for 21 days.

**Groups**	**Female**			**Male**		
	**HSI**	**BSI**	**GSI**	**HSI**	**BSI**	**GSI**
**Control**	2.90±0.94	1.10±0.40	9.20±1.70	1.00±0.50	1.58±0.65	0.81±0.26
**Solvent Control**	2.70±0.85	1.50±0.25	9.70±2.20	1.40±0.36	1.78±0.64	0.88±0.17
**0.4mg/L TEB**	2.30±0.67	1.6±0.60	7.27±1.70	2.54±0.97*	1.63±0.35	0.78±0.18
**0.8mg/L TEB**	3.50±0.98	1.40±0.50	7.40±1.60	2.27±0.60*	1.73±0.56	0.94±0.26
**1.6mg/L TEB**	3.80±1.07*	1.40±0.66	6.55±1.53*	2.40±0.99*	1.93±0.75	0.83±0.21

*$P < 0.05$ indicates a significant difference between the exposure and control groups; the data are expressed as the mean ± SEM.

Table S2. Expression of the genes associated on social behavior and HPG axis of female zebrafish exposed to TEB.

Organs	Genes	0	DMSO	0.4mg/L TEB	0.8mg/L TEB	1.6mg/L TEB
Brain	*gnrh2*	1.04±0.08	0.98±0.02	0.59±0.06	0.64±0.08	0.44±0.03*
	gnrh3	1.19±0.07	0.93±0.07	1.20±0.10	0.92±0.08	0.85±0.00
	fshβ	1.17±0.11	1.24±0.08	1.25±0.03	0.99±0.10	1.36±0.13
	gnrhr3	0.99±0.02	1.55±0.07	0.84±0.05	0.45±0.00*	0.51±0.01*
	avp	1.06±0.03	1.24±0.03	0.66±0.07	0.39±0.04*	0.32±0.04*
	oxt	1.01±0.03	1.13±0.06	0.48±0.01	0.47±0.04	0.37±0.04*
	scg2a	1.04±0.03	1.22±0.03	0.89±0.10	0.55±0.06	0.55±0.02
	scg2b	0.98±0.02	1.07±0.15	1.23±0.14	0.51±0.04	0.45±0.00*
	Lhβ	1.00±0.03	0.82±0.02	0.54±0.02	1.28±0.06	0.43±0.00*
Liver	*vtg1*	1.03±0.07	1.15±0.03	0.63±0.06	1.21±0.09	1.29±0.05
	vtg3	1.01±0.02	0.92±0.05	0.64±0.05	0.58±0.12	0.43±0.01*
	era	1.07±0.03	1.06±0.04	0.71±0.02	0.63±0.03	0.43±0.00*
	erβ	1.01±0.03	0.95±0.00	0.52±0.02	0.66±0.01	0.52±0.04
Ovary	*star*	1.03±0.02	1.00±0.01	0.66±0.07	1.20±0.09	2.32±0.15*
	cyp11a	1.14±0.00	1.04±0.01	1.01±0.06	1.64±0.01*	1.54±0.03*
	cyp19a	1.08±0.04	1.07±0.03	1.27±0.07	0.54±0.02	0.37±0.04*
	17β-hsd	1.01±0.01	1.11±0.16	0.65±0.16	0.42±0.05	0.43±0.01*
	fshr	1.01±0.06	1.18±0.02	0.52±0.02	0.60±0.02	0.31±0.03*
	lhr	1.00±0.04	1.25±0.08	0.73±0.17	0.9±0.11	0.54±0.09

era	0.98±0.03	1.09±0.02	0.59±0.03*	0.56±0.00*	0.55±0.03*
cyp17	1.00±0.02	1.04±0.05	0.67±0.07	0.49±0.01*	0.60±0.01

*$P < 0.05$ indicates a significant difference between the exposure and control groups; the data are expressed as the mean ± SEM.

Table S3. Expression of the genes associated on social behavior and HPG axis of male zebrafish exposed to TEB.

Organs	genes	0	DMSO	0.4mg/L TEB	0.8mg/L TEB	1.6mg/L TEB
Brain	*gnrh2*	0.99±0.03	0.98±0.04	1.09±0.03	0.61±0.03	0.84±0.04
	gnrh3	1.02±0.06	1.14±0.06	1.14±0.12	0.67±0.12	0.82±0.07
	fshβ	1.02±0.06	1.11±0.04	1.04±0.04	0.95±0.00	0.73±0.10
	gnrhr3	1.00±0.02	1.04±0.06	0.90±0.06	0.69±0.03	0.50±0.03*
	avp	1.05±0.03	1.24±0.04	1.21±0.04	0.79±0.10	1.15±0.07
	oxt	1.15±0.03	1.15±0.03	1.32±0.05	1.14±0.04	0.42±0.02*
	scg2a	1.04±0.04	1.12±0.04	1.12±0.09	0.64±0.07	0.77±0.07
	scg2b	1.01±0.04	0.71±0.01	0.89±0.07	0.601±0.07	0.82±0.10
	lhβ	1.02±0.02	1.01±0.11	1.10±0.18	3.04±0.15*	2.26±0.04*
Liver	*vtg1*	1.00±0.03	1.03±0.02	1.06±0.03	1.11±0.00	0.80±0.03
	vtg3	1.01±0.05	0.98±0.04	0.61±0.02	0.73±0.15	0.47±0.03*
	era	1.03±0.05	1.02±0.09	1.59±0.14	1.59±0.09	1.57±0.23
	erβ	1.03±0.05	1.06±0.06	1.04±0.08	1.06±0.12	0.32±0.06*
Testis	*star*	1.03±0.06	1.06±0.02	1.05±0.01	0.90±0.04	0.43±0.04*
	cyp11a	1.04±0.00	0.90±0.03	0.97±0.05	1.26±0.09	0.66±0.08

cyp19a	1.00±0.04	0.90±0.00	0.95±0.08	0.77±0.17	1.14±0.07
17β-hsd	1.01±0.02	1.27±0.04	1.31±0.08	1.35±0.14	0.90±0.10
fshr	1.01±0.03	1.16±0.07	1.09±0.06	1.00±0.14	0.49±0.05*
lhr	1.09±0.06	1.16±0.03	0.50±0.02*	0.55±0.07*	0.32±0.04*
era	1.03±0.01	1.02±0.10	1.02±0.10	0.93±0.10	0.36±0.02*

*$P < 0.05$ indicates a significant difference between the exposure and control groups; the data are expressed as the mean ± SEM.

Table S4. The full name of the genes.

Organs	genes	Gene names
Brain	*gnrh2*	*Gonadotropin releasing hormone 2*
	gnrh3	*Gonadotropinreleasing hormone 3*
	fshβ	*Follicle stimulating hormone beta*
	gnrhr3	*Gonadotropinreleasing hormone receptor 3*
	avp	*Vasotocin*
	oxt	*Isotocin*
	scg2a	*Secretogranin-IIa*
	scg2b	*Secretogranin-IIb*
	lhβ	*Luteinizing hormone beta*
Liver	*vtg1*	*Vitellogenin 1*
	vtg3	*Vitellogenin 3*
	era	*Estrogen receptor a*

	erβ	*Estrogen receptor β*
	star	*Steroidogenic actue regulatory protein*
	cyp11a	*cytochrome p450 aide-chain cleavage*
	cyp19a	*cytochrome p450 aromatase*
gonads	*17β-hsd*	17*β-hydroxysteroid dehydrogenase*
	fshr	*Follicle stimulating hormone receptor*
	lhr	*Luteinizing hormone receptor*
	era	*Estrogen receptor a*
